# Supplementary material for: Are carbon emissions trading and green financial instruments synergistic? -Comprehensive quantitative research based on content analysis
Source: PLoS One. 2024 Mar 7;19(3):e0298601. doi: 10.1371/journal.pone.0298601 (PMC10919601; doi:10.1371/journal.pone.0298601)
Supplement: S1 Data — (ZIP) [file pone.0298601.s001.zip › Data/地级市Z_F工作报告.zip!/╜¡╦╒/│ú╓▌╩╨/└┤╫╘╣1⁄2╓┌║┼í╛┬φ┐╦╩2╛▌═°í┐_2005.docx]

各位代表：

　　现在，我代表常州市人民政府，向大会作工作报告，请予审议，并请市政协各位委员和其他列席人员提出意见。

　　一、2004年工作回顾

　　过去的一年，是我市发展进程中很不寻常的一年。在中共常州市委的正确领导下，全市人民以邓小平理论和“三个代表”重要思想为指导，深入贯彻党的十六届三中、四中全会精神，认真落实科学发展观和中央宏观调控的决策部署，紧紧围绕“两个率先”，努力推进经济社会协调可持续发展，取得了改革开放和现代化建设的新成就。

　　———经济实力明显提高。预计，全市地区生产总值达到1100亿元左右，按可比价增长15.5%，人均GDP超过3800美元，这标志着我市经济社会发展上了一个新平台。财政总收入实现175.54亿元，增长28.98%，其中地方一般预算收入67.34亿元，增长32.55%。完成全社会固定资产投资580亿元以上，增幅达到30%，其中工业投入340亿元，增长35%左右。农业总产值增长9.3%，粮食生产实现单产、总产和效益全面增长，水稻单产居全省第一。规模以上工业现价产值达到2013亿元，增长31.8%。高新技术产业产值占全部工业的比重达到28%左右，比上年提高约2个百分点。服务业增加值增长14.1%，全社会消费品零售总额增长15.5%，金融机构存贷款余额分别比年初增长16.4%和21.6%，接待国内外游客1000万人次。

　　———发展活力明显增强。民营经济已经成为我市的主导经济，全年新发展私营企业8586户，总数达3.8万户，户均注册资本114万元。民营企业对工业增长的贡献份额达到72.6%。规模和品牌效应逐步显现，产值超过10亿元的企业达23家，新科、中天、常林等三家企业集团年销售收入均超过60亿元，上上电缆等4只产品获“中国名牌产品”称号。各项改革进一步深化和完善，解决了一批企业改制和对外合作中的突出问题，完成了12家生产经营类事业单位的改制转企。

　　———开放水平明显提升。全年协议注册外资和实际到账外资分别达到22.2亿美元和6.5亿美元，增长8%和30%。项目规模和领域实现新突破，全年新签千万美元以上项目129个，德州仪器、圣戈班、沃尔玛、欧尚等世界“500强”企业和纳科晶圆、和美液晶等高科技大项目落户我市，博世力士乐等跨国公司增资扩股，市政、教育等对外合作也迈出了新步伐。全年进出口总额实现68亿美元，增长30%，其中出口46亿美元，增长30%，机电产品占出口总量50%左右。外经营业额达到2亿美元，增长26.3%。沿江开发加快推进，常州港外贸货物吞吐量增长20.4%。

　　———社会事业明显进步。在综合实力百强城市评选中，我市社会发展指标位居全国第20位，名列全省第一。被列为国家首批知识产权试点城市，连续三届获得“全国科技进步先进城市”称号。新增省级以上高新技术企业47家、高新技术产品129只，国家级特色产业基地累计达到6个，专利申请量达1653件。编制完成市区教育布局规划；常州高职教育基地西区基本建成，在校学生达到4万名；北郊中学新北校区等一批教育基础设施落成；高考万人进线率和职校单招本科录取率继续名列全省第一。首次举办“金秋人才活动周”，全年引进各类专业人才1.46万名。成功举办中国国际（常州）卡通·数码艺术周暨第三届常州艺术节，完成市区有线电视宽带网双向改造，编制了前后北岸历史文化保护街区规划。进一步完善公共卫生体系，规划和建设市妇幼保健院病房楼等10个卫生基础设施项目。连续8年被评为全国全民健身先进城市，我市运动员在第12届残奥会上取得“两金一银”和破一项世界纪录的优异成绩。

　　———城建力度明显加大。获得“国家环保模范城市”命名授牌，通过省级园林城市考核验收。交通、城市基础设施建设加快推进。宁杭、常澄高速常州段建成通车，沪宁高速扩容和宁常、扬溧高速建设进展顺利，境内高速公路通车里程突破100公里。金武公路竣工通车，建成农村公路1640公里，在全省率先实现村村通公路的目标。运河南移、312国道改线和沿江基础设施建设有序推进。完成城市水环境整治一期工程，开工建设横塘河北枢纽防洪工程。东方大道、常戚路等一批城市道路工程相继完成，积极推进常澄路—万福路综合改造，青洋路南段、长江路一期等南北干道建设进展顺利，城市生活垃圾填埋场二期工程基本建成，南大街区成片改造全面竣工。电力、电信和城市信息化设施的建设力度不断加大。荆川公园扩建敞开，建成武进区市民广场、政成桥等一批公共绿地和道路景观，新增城市绿地522.5公顷。完成新一轮城市总体规划和历史文化名城保护等各类专项规划修编，城市管理工作得到加强。

　　———人民生活明显改善。城镇居民人均可支配收入和农民人均纯收入居全省第三位，达到12867元和6280元，分别增长13.8%和13%。全市城乡人均储蓄20759元，增长15.4%，个人消费贷款、住房公积金贷款分别超过100亿元和10亿元。建成经济适用房10万平方米，整治老小区250万平方米，荣获“中国人居环境范例奖”。企业离退休人员的养老金和企业指导工资标准分别有了增加和提高。预计全年新增就业13万人，下岗失业人员再就业2.4万人，城镇登记失业率控制在3.6%以内。

　　各位代表，过去的一年，全市上下经受了比以往更严峻的考验，付出了比以往更多的心血和汗水，成绩来之不易。这充分显示了全市人民不畏艰难、开拓创新的精神风貌，必将进一步激发全市人民奋发向上的信心和力量！

　　回顾一年来的工作，我们的主要做法和体会是：

　　（一）坚持以科学发展观统揽全局。我们把落实科学发展观与加快“两个率先”相结合，与做好人口、资源、环境工作相结合，与深化改革、调整结构相结合，与执政为民、富民优先相结合，与规范政府行为、依法行政相结合。去年以来，宏观环境的变化和铁本项目的查处，使我们发展中的困难和矛盾更加突出。面对新的形势，我们坚持统一思想、服从大局，坚决、认真地贯彻落实宏观调控的各项决策，深入进行理性思考，把宏观调控作为调整经济结构、转变增长方式的新机遇；积极、妥善地处理好铁本项目的善后及重组工作，尽可能降低损失、消除影响，保护好、引导好、发挥好各方面的积极性，保持社会稳定的良好局面；全面、准确地把握科学发展观的精神实质，把发展热情和科学态度统一起来，协调推进各项工作，促进经济又快又好发展。同时，按照中央和省的统一部署，开展了土地市场秩序专项治理和投资项目清理。

　　（二）坚持创新发展理念和思路。理念决定思路，思路决定出路。一年来，我们在深入基层现场办公和调查研究的基础上，坚持从常州实际出发，以创新的理念谋划发展，以创新的思路增创优势。在发展定位上，明确提出作为中心城区的天宁、钟楼要大力发展现代城市经济，成为我市商贸、金融和文化中心；新北区要成为高新技术示范区和现代化新城区；武进区要成为现代科教区、高新技术集聚区和生态居住区；戚区要按照做优、做精、做强东大门的要求加快发展；金坛市要成为全国出口服装制造名城和盐化工产业基地；溧阳市要成为苏浙皖边界的中心城市和山水型、资源型、生态型的旅游名城。在产业升级上，坚持二三产业相互支撑、协调发展，把发展服务业摆上更加突出的位置。在结构调整上，提出“控制压缩部分行业，改造提升传统产业，做大做强机电产业，加快培育新兴产业”的要求，制订了制造业升级三年行动纲要。在增长方式上，进一步明确依靠科技进步的指导思想，启动实施科技经费“三年倍增”和高新技术产业产值“三倍增”计划，不断增强经济持续发展能力和竞争力。

　　（三）坚持用新的机制和办法破解发展难题。面临困难多、挑战多、矛盾多的新情况，我们坚持用新思路、新机制、新办法解决经济生活中的突出问题。一是以集约发展破解用地难题。积极引导和推进企业集群、产业集聚、资源集约利用，大力推进标准厂房建设，明确外资低于500万美元、内资低于3000万人民币的项目原则上都进标准厂房，千方百计提高投入产出强度。二是依靠市场主体和机制创新，努力解决资金紧缺问题。发挥常州投资集团、交通产业集团、城市建设集团、民防投资公司等投融资主体的作用，通过运作资源筹措城市建设资金。引导企业采取股权转让、资本运作等方式解决资金难题。三是通过调整结构，确保投资稳定增长。准确理解、把握中央宏观调控政策，及时调整年初确定的投资项目计划，加大对高新技术产业、农业、服务业和基础设施的投入，其中高新技术产业投资增长44%。四是因势利导，着力推进招商引资。确立“展示产业、突出招商、宣传常州、促进发展”的主题，放大国际中小企业商品博览会的品牌效应，利用外资取得了新成效。努力发挥民营企业在扩大对外开放中的优势，首次大规模地组织民营企业出国招商，使民营经济成为我市招商引资的重要载体。

　　（四）坚持把富民放在突出位置。我们始终坚持执政为民、富民优先，把更多的时间和精力用在解决基层群众的实际问题和制定、落实富民政策上，把更多的财力和物力用在经济社会协调发展和提高人民生活水平上。一是认真落实中央和省扶持农业的一系列政策，先后出台了“一免三补”等政策措施，全年免征农业税及附加和发放直补资金共1.5亿元，农民人均受益70元。提前一年基本完成草危房改造、改水、农村公路建设、新型合作医疗和税费改革等农村五件实事，有效地改善了农民的生产生活条件。二是制定出台服务民营企业的28条措施，引导民营经济加快制度创新和科技进步，使民营经济成为增加城乡居民收入的重要渠道。三是不断完善社会保障体系，着力推进社保扩面，全年新增参加养老保险20.94万人、医疗保险17.64万人，实现了年增参保人数的历史性突破，还出台了低保人员医疗救助办法，切实保障人民群众的根本利益。

　　（五）坚持不断提高政府领导发展的能力和水平。一年来，我们坚持“有所为、有所不为”，着力增强政府驾驭市场经济、促进社会发展的本领，努力办成一些想办而一时难办的实事、好事。一是进一步转变政府职能，通过强化规划定位、配置资源、培育主体、制定政策和依法监管，提高对经济社会发展的调控能力，求真务实谋划发展。二是注重发挥市场机制和政策引导作用。继续加大城建、交通领域资源的市场化运作力度，并将这一理念运用到社会事业方面，规划建设了市博物馆新馆等一批项目。通过创新经济适用房的运作机制，统筹解决困难群体住房和老小区整治、落实私房政策所需资金等问题。制定并落实支持高新技术产业、加快软件园发展等政策措施，有力地促进了科技创新和软件产业发展。三是坚持依法行政。我们以实施行政许可法为契机，不断规范和创新政府行政行为，制定了《常州市人民政府工作规则》，进一步建立和完善科学的决策程序和机制，深化行政执法体制改革，完善相对集中行政处罚权，告知承诺制、集中联合年检年审制等取得新成效。四是切实维护社会稳定。出台新的城市房屋拆迁管理和被征地农民基本生活保障办法，开展被征地农民补偿安置费清欠专项治理；尽力解决企事业单位改制中的突出矛盾和问题，保障职工的合法权益；建立大调解机制，及时处理群众来信来访。高度重视安全生产，大力推进“平安常州”建设，社会治安综合治理取得新的成效。

　　与此同时，全市各级政府切实加强精神文明建设，进一步做好“双拥”、国防动员、民兵预备役和人防工作，革命史馆和国防园相继竣工。高度重视依法治市和民主法制建设，加强廉政建设和反腐败斗争。深入开展纠风和专项治理工作，继续整顿与规范市场经济秩序。认真做好新闻出版、文物保护、外事、侨务、监察、审计、统计、档案、民族和宗教等工作。人口与计划生育、妇女儿童、老龄、残疾人等各项事业都取得了新成绩。

　　各位代表，去年我们所取得的每一项成绩，是全市人民迎难而上、负重奋进的结果，社会各界、各方面都作出了积极努力。我代表市人民政府，向全市广大工人、农民、知识分子和离退休老同志，向人大代表、政协委员、各民主党派、工商联、各人民团体、无党派及各界人士，向驻常部队指战员、武警官兵和部、省驻常单位，向所有关心、支持、参与常州发展的海内外朋友们，表示衷心的感谢！

　　在充分肯定成绩的同时，我们也清醒地看到工作中存在的问题和不足，主要是经济结构性矛盾仍然突出，经济增长方式有待转变，对外开放工作还要进一步加强，基础设施建设任务还很繁重，人口、资源、环境的压力较大，就业和社会保障工作仍需进一步强化，农民增收的长效机制还未真正形成，影响社会稳定的因素依然较多。同时，机关作风和公务员队伍建设需要进一步加强，发展理念还需进一步创新，创业环境有待进一步优化。我们一定会高度重视这些问题，切实采取措施加以解决。

　　二、2005年工作安排

　　今年是完成“十五”计划的决战之年，也是我市推进科学发展、实现全面小康的关键之年。纵观国内外形势，仍然是机遇和挑战并存。一方面，随着科学发展观和宏观调控政策措施的落实，国内经济运行中不健康不稳定的因素得到有效抑制；国际产业和资本向“长三角”地区转移的势头依然强劲；我市经济社会发展的基础更加坚实，思路更加清晰，经济自主增长活力显著增强。另一方面，世界经济增长存在诸多不确定因素，中央实施“双稳健”的宏观调控政策，我市发展中长期积累、制约全局的深层次矛盾和问题将逐步显现，周边城市的竞争压力日益加大。我们必须进一步增强危机意识、责任意识和发展意识，以超常规的思路、机制和方法，开创率先发展、科学发展的新局面。

　　今年政府工作的指导思想和总体要求是：以邓小平理论和“三个代表”重要思想为指导，深入贯彻党的十六届三中、四中全会精神，全面落实科学发展观，紧紧围绕“两个率先”，按照市委九届十二次会议确定的“六个全力推进”的要求，进一步解放思想、创新理念、抢抓机遇，以转变经济增长方式为根本途径，加快推进改革开放，加快调整经济结构，加快城市化建设步伐，加快构建和谐社会，加快富民进程，促进常州经济社会又快又好地发展。

　　2005年经济社会发展的主要预期目标是：地区生产总值增长13%，提前1年实现地区生产总值达到1200亿元的目标；地方一般预算收入增长15%；全社会固定资产投资增长25%以上；外贸出口增长20%，协议注册外资和实际到账外资分别增长30%以上；全社会消费品零售总额增长13%，城镇居民人均可支配收入和农民人均纯收入分别增长10%和8%以上，城镇登记失业率控制在4%以内，人口自然增长率控制在1‰以内。

　　为完成上述目标，我们将着重抓好以下八项工作：

　　（一）加大对“三农”的支持力度，保持农村经济稳定增长

　　坚持把解决好“三农”问题作为实现全面小康的重中之重，进一步加大农村改革力度，完善和强化对农业的扶持政策，调整农业和农村经济结构，着力提高农业综合生产能力，切实加快富裕农民的进程。

　　加快发展现代农业。按照我市现代农业产业发展总体规划，在稳定粮食种植面积的基础上，进一步推进农业结构的战略性调整，发展“优质、高效、安全、生态”农业。发挥我市农业资源丰富的优势，加快推进花卉苗木、特种水产、优质稻米、经济林果、食草畜禽等优势产业带区建设。推进农业科技园、农业标准化示范区和农技推广体系建设，发展无公害农业，做好农产品检测工作。鼓励“三资”投资开发农业，推进溧阳现代农业示范园、金坛丘陵山区畜牧业等重点项目建设，积极发展农业产业化经营。继续举办好常州名优农产品上海交易会，加大农产品市场开发和品牌建设力度。

　　不断深化农村各项改革。加快推进农村集体经济股份制改革，量化集体资产，让农民持股分红；积极推进农村土地股份制改革，完善并落实“土地换保障”制度，尽快建立土地承包经营和流转收益机制，增加农民的资产性收入。大力发展农民专业合作经济组织，提高农民经营的市场化组织程度。继续深化农村税费改革，全面落实好农村各项政策，已经实行的扶农政策不变，已经给农民的实惠不减。加大工业反哺农业、城市支持农村的力度，加快农村劳动力转移，不断提高农民的非农收入。

　　增强县域经济实力。按照建设中等城市的目标要求，编制和完善辖市新一轮城市总体规划和专项规划。大力推进基础设施建设，加快规划建设常溧高速公路，尽早实现市到辖市互通高速的目标；开工建设241省道天目湖段，完成金宜线续建工程，推进县乡公路建设，形成与过境高速公路相配套的道路网络。进一步加强小城镇的规划编制，增强文化内涵和特色风貌，建设一批具有示范和带动效应的重点中心镇。加快工业化进程，协调三次产业发展。充分发挥资源优势和产业优势，提升机电、服装、食品、建筑安装等特色产业水平，壮大精细化工、新型材料等新兴产业规模，发展山水经济、绿色经济，大力提升县域经济水平。

　　（二）强化科技创新，促进制造业结构优化升级

　　全面实施制造业升级三年行动纲要，把加快科技进步、发展高新技术产业作为调整优化产业结构的切入点和突破口，切实转变经济增长方式，走新型工业化道路。

　　大力发展高新技术产业。积极实施科技经费“三年倍增”计划，加快推进高新技术产业产值“三倍增”，力争今年高新技术产业产值占全市工业的比重达到30%以上。加大纳科晶圆、德州仪器、和美液晶、莫本通讯等重大项目的推进力度，扶持发展新科、远宇等骨干企业，加快构筑以电子信息设备制造业为龙头、集成电路产业为核心、电子元器件为配套、软件及信息服务业为支撑的发展新格局。推进圣戈班、安德利等新材料项目的建设，放大涂料、稀土和新型纺织材料等产业优势，发展高性能基础材料、光电材料和新能源材料等产业。大力发展新医药，抓好千红、方圆、四药等企业的高科技成果转化项目，努力培育一批具有自主知识产权和创新能力的“三药”科技企业。

　　改造提升优势产业和传统产业。进一步做大做强机电产业。发挥戚机厂、戚研所的产品、技术和人才优势，加快与跨国公司的合作，力争使我市成为轨道交通产业基地；利用我市变压器、电线电缆、输变电控制设备的良好产业基础，推进常变与东芝的全面合作，努力形成我市输变电产业的新优势；依托常林集团的带动辐射，扩大对外合作，加快韩国现代工业园建设，进一步增强我市工程机械的产业特色；以常柴、常发等骨干企业为龙头，加快技术更新和产品升级，提升我市动力机械产业的发展水平；发挥我市数控机床等基础优势，主动吸引外资、技术和品牌，加快装备制造业的发展步伐。积极运用高新技术和先进适用技术，提升纺织服装、塑料包装等传统产业。深入实施“制造业信息化工程”，争取市级以上制造业信息化试点企业达到80家。加大品牌培育力度，力争新增中国名牌产品2只、中国驰名商标1只。与此同时，加快培育和发展大企业大集团，力争实现销售超百亿企业的新突破。

　　加大工业有效投入。坚持能快则快、有保有压、优化结构，继续加大有效投入，培育新的经济增长点。通过利用金融资本、海外资本、民间资本和开展资产资本运作，确保工业投入总量再上新台阶，全年实施98个超亿元项目，工业投入突破400亿元。不断优化投资结构，确保戚电厂燃气发电机组年内竣工，加快常州电厂一期工程、5万吨精密铜管、真空制盐等重点项目建设。鼓励企业积极引进国际先进技术装备，努力提高投入产出水平。同时，大力发展循环经济，严格控制高耗能、高耗水、高污染的项目，防止盲目投资和低水平重复建设。

　　（三）着力提升对外开放水平，加快经济国际化进程

　　今年我国将进入加入世贸组织的后过渡期。我们要积极应对、趋利避害，把发展开放型经济作为调整产业结构、转变经济增长方式最现实、最重要的途径，全方位、宽领域、高层次提高对外开放水平。

　　全力推进招商引资。把招商引资作为今年经济工作的首要任务。加大对外宣传力度，以

日韩、欧美为重点，每月组织大规模的宣传推介活动，不断提升常州在国际上的影响力和知名度。营造浓厚的招商氛围，增强招商引资的紧迫感、责任感，各级领导干部要身先士卒，既当指挥员，又当战斗员，出国出访都要突出招商引资主题。全力以赴主攻外资大项目、新项目，充分利用我市电子信息、机电装备、车辆制造等产业优势，找准与国际大公司产业转移的结合点，着力引进辐射力强、产业链长的超亿美元项目；加快沿江开发步伐，抓好出口加工区的批办与建设，完善配套设施功能，大力引进一批龙头型、基地型企业；加强对现有外资企业特别是跨国公司投资项目的服务，在沟通中亲商、在服务中安商、在支持中富商，不断扩大合作范围和领域，促进增资扩股、以外引外。加快招商机制的创新，用市场化运作的办法，健全报酬与业绩直接挂钩的招商激励机制，建立一支数量足、业务精的招商队伍。同时，继续发挥国际中小企业商品博览会的品牌效应，扩大对外交流，推进招商引资。

　　加快开发园区建设。加强规划和产业引导，进一步明确开发园区的功能定位。常州高新区要大力发展电子信息及软件、“三药”等高新技术产业和工程机械等优势产业，武进高新区要重点发展机械装备工业、微电子等新兴产业，天宁开发区要积极培育高档纺织面料、服装和机电产业，钟楼开发区要加快壮大电子信息、新材料、精密机械等三大产业，戚墅堰开发区要加快形成轨道交通、汽车配件的产业优势，其他省级开发区也要进一步明确发展方向，努力形成自身特色。加大市场化运作力度，加快基础设施建设，不断提高开发园区的承载能力。集约利用土地资源，制定出台有关政策措施，鼓励建设多层标准厂房，全年完成300万平方米的建设任务，省级以上开发区投入和产出增幅都要达到50%以上。按照市场配置资源的原则，倡导联合开发，拓展园区发展空间。围绕“小政府大社会、小机构大服务”的要求，推进“扎口收费、一站式服务”，创新管理体制和工作机制，形成精干高效的管理体系。

　　积极扩大对外贸易。深入实施科技兴贸、以质取胜战略，切实转变外贸增长方式，鼓励扩大一般贸易，引导加工贸易转型升级。着力优化出口产品结构，进一步提高机电设备和高新技术产品的出口比重。鼓励发展面向中小企业的外贸业务咨询、代理服务、法律支持等中介机构，支持企业到境外参展和进行国际认证。积极申报保税物流中心，加快建设海关、国检溧阳办事处，申报武进办事处，推进“大通关”建设。同时，支持企业“走出去”拓展市场空间。

　　（四）积极培育和扩大内需，提升服务业发展水平

　　加快发展服务业是优化产业结构、促进制造业升级的重要支撑，是完善城市功能、扩大消费需求的内在要求，是增加就业岗位、改善生活质量的有效途径。今年我们要进一步加快服务业发展，不断提高服务业增加值占生产总值的比重，提高服务业从业人员占全社会从业人员的比重。

　　进一步扩大消费需求。抓住经济增长、消费升级、市场活跃的有利时机，加快发展文化、体育、社区服务等消费需求潜力大的行业，积极培育汽车、住房、信息服务和非义务教育等消费热点。抓好凌家塘、长江塑化、江南机电、南田购物广场等市场改造和建设，进一步集聚人流、物流、信息流，形成新的消费增长点。实施商业网点规划，加快布局合理调整，进一步提升商贸、餐饮等行业的服务水准，增强区域中心城市的消费集聚作用。努力开拓农村市场，完善农村流通体系，提高农村消费水平。

　　加快发展新型服务业。充分利用我市制造业优势，积极培育工业设计、技术中介、法律咨询等新兴服务行业；依托港口、机场、高速公路等综合运输优势，加快现代物流体系建设；不断完善劳动力、产权等要素市场，增强服务业的生产服务功能。积极推进旅游规划的实施，加快培育旅游投资开发主体，通过市场化运作，推进太湖湾旅游度假区一期、淹城森林公园二期等重点项目建设。认真开展“旅游宣传促销年”活动，不断拓展旅游市场，全年实现旅游总收入增长20%左右。

　　推进服务业对外开放。抓住国际服务贸易转移和CEPA带来的机遇，大力吸引外资、港资投向服务业。积极利用外资提升商贸业态，推进跨国商贸企业在常项目的建设，推广大型连锁、集中配送、电子商务等现代流通方式。争取国内外金融保险机构来常设立营业网点，进一步提升我市金融发展水平。举办好2005年常州服务业招商会，推出一批市场前景好、投资规模大的服务业项目，鼓励外资、民资进入教育、卫生和市政公用事业等领域。

　　（五）深化市场取向改革，增强经济社会发展动力

　　围绕建立活力强、效率高的经济运行体系，不失时机地加快推进各项改革，营造体制机制新优势，为经济发展提供强大动力。

　　积极推进各项改革。按照“有利于维护职工权益、有利于促进企业发展、有利于降低政府改制成本”的原则，坚持改革转制与扩大对外开放、引进战略投资相结合，与城市规划和整合资源相结合，继续深化国有集体企业改革，做好企业股权结构调整、职工身份置换等工作，健全法人治理结构，建立现代企业制度；完成生产经营类事业单位的改制转企，加快行政管理类和社会公益类事业单位的改革。按照权利、义务、责任相统一，管资产、管人、管事相结合的原则，建立新的国有资产管理体系，促进国有资产保值增值。加快投融资体制机制改革，推动资本市场进一步开放和发展。完善市、区财政体制改革，强化税收征管；加强部门预算、政府采购和收支两条线管理，在市本级全面推行国库集中支付制度，进一步健全公共财政体系。大力建设信用监督和信用服务体系，提高全社会信用水平。切实加强行业协会、同业商会建设，规范发展中介机构，充分发挥他们在经济和社会发展中的作用。

　　提升民营经济发展水平。按照民营经济总量“三年翻番”的目标要求，确保全年新增注册资本超过100亿元。认真落实促进民营经济发展的各项政策措施，为民营经济发展创造一视同仁的法制环境、政策环境和市场环境，进一步增强民营企业的发展信心。有计划、有步骤地推动100家民营企业完善法人治理结构，建立现代企业制度，实现传统管理向现代管理的转变。引导民营企业走科技创新之路，加大技改投入，加快技术积累，提高产品科技含量和企业的核心竞争力。支持民营企业走资本运作之路，通过并购重组、出让股权、上市发行股票债券等途径，吸引各类资本，促进结构优化和规模效益的提高。鼓励民营企业走对外开放之路，加快推进合资合作，在开放中做大做强。同时，切实解决市场准入、担保体系建设等难题，促进民营中小企业向工业集中区发展，加强民营企业家的培训服务，引导民营企业依法规范经营。

　　（六）加快基础设施建设，进一步提升城市品位和功能

　　以创建国家园林城市和生态城市为目标，进一步创新规划、建设和管理机制，加大基础设施投入力度，优化配置城市资源，加强综合环境建设，构筑经济社会发展的更高平台。

　　加快构建城市基础设施体系。深入实施大交通战略，继续推进沪宁高速扩容和宁常、扬溧等高速公路建设；积极做好西绕城高速的前期工作，力争早日开工建设；加快推进312国道常州段、省道239和338等改扩建工程，争取年内建成通车；按计划推进运河南移和沿江基础设施建设；建设凌家塘、武进客运服务中心。进一步完善城市功能，重点实施沟通南北的长江路北段、青洋路南段以及常澄路—万福路、常焦路、丽华南路、外环路改造等道路工程；加大城市公用设施建设，建成江边污水处理厂一期、天然气利用二期等项目。

　　创建优美的人居环境。按照创建国家园林城市的目标，推进园林绿化十大工程，新增生态绿地650公顷。继续推进一批道路绿化景观工程和街头小游园建设，搞好沪宁高速、常澄高速等道路两侧“绿色通道”建设；积极实施环境保护和建设三年纲要，开展“全国生态示范区”建设，推进印染企业提标排放、禁用高污染燃料和市区部分污染企业搬迁；开工建设后塘河等10条市河的水环境整治工程。继续完善城市防洪体系建设。抓好“城中村”的综合改造试点，加快旧城区成片改造步伐。完善“两级政府、三级管理”体制，强化街道、社区建设和管理，建立“大城管”机制，深化市容环境、交通秩序综合整治，着力解决摩托车、马路市场和违章建筑等群众反映强烈的问题。以建设常州“西太湖”为目标，加快制定滆湖中长期整治开发规划，启动清淤、治污等治理工程。

　　管理和配置好城市资源。充分发挥规划对城市资源的调控作用，积极开展制造业、现代服务业、城市建筑空间布局和南北“两翼”的规划研究，抓紧编制分区规划、城市街景和城市轨道交通等一批规划。做好新一轮土地利用总体规划编制，调整基本农田保护区规划，不断提升城市资源价值。切实管理和配置好土地资源，加大土地收购储备力度，垄断土地一级市场，搞活土地二级市场，严格执行经营性用地招标、拍卖和挂牌交易制度。提高项目管理水平，通过市场化竞争、规范建设程序、控制工程造价和加强审计监督，努力降低建设成本。加快城市道路、市政公用事业市场化改革的力度，出让供水、污水处理等行业的部分经营权，实施生活垃圾焚烧发电BOT项目和市政工程BT项目，提高城市资源的运行质量和整体效益。

　　（七）统筹经济社会发展，努力构建和谐社会

　　坚持以人为本，维护好、发展好人民群众的现实利益和长远利益，不断完善就业与社会保障体系，协调发展各项社会事业，促进物质文明、精神文明与政治文明共同进步。

　　高度重视就业和社会保障工作。认真落实税费减免、小额担保贷款等政策措施，进一步健全劳动力市场和信息网络，加强对城镇下岗失业人员等困难群体的就业援助和技能培训。继续把社保扩面作为事关全局的一件大事，建立“一把手”负总责的责任制和部门协调联动机制，确保今年养老保险净增5万人、医疗保险净增5.5万人。积极推进农村基本养老保险，巩固和扩大以大病统筹为主的新型合作医疗制度，继续做好住房公积金的扩面工作。合理调整分配关系，完善最低生活保障制度，提高城乡低保、城镇“三无”和农村“五保”对象的供养标准，建立与经济发展相适应的增长机制。

　　大力发展各项社会事业。积极实施科教兴市战略，推进特色产业基地建设，新建市级以上工程技术研究中心5家、企业技术中心15家，抓好国产网络计算机等一批产学研合作和成果转化项目。加快教育资源的优化整合，开展新一轮中小学布局调整，全面落实“以县为主”的农村义务教育管理体制；启动新北职教中心建设，实施老年大学搬迁扩容；大力发展高教事业，加快推进高职教育基地东区建设。进一步优化人才结构，加快培养、引进制造业急需人才，吸引更多的境内外高层次人才，努力构筑人才高地。推进公共卫生监督、疾病预防控制体系建设，加强农村医疗卫生工作，开工建设市传染病医院综合大楼、市中心血站等一批重点项目。大力繁荣和发展文化事业，积极推进文化体制改革、文化资源整合和文化产业发展，抓好国家动画产业基地和博物馆新馆、规划展示馆、少儿图书馆等设施建设，加强文物保护工作，创出一批文艺精品工程，办好’2005国际（常州）卡通·数码艺术周。深入推进社区文化建设，不断丰富基层群众生活。加快体育设施建设，承办好全国十运会常州赛区的各项赛事，积极备战第16届省运会，全力申办第17届省运会。深入推进精神文明和民主法制建设，大力弘扬常州市民精神，加强未成年人思想道德建设，深入开展学习型城市和文明城市创建活动。创新人口与计划生育工作机制，加强流动人口管理，稳定低生育水平。加强国防动员和民兵预备役工作，积极做好民政、外事、侨务、档案、新闻出版、民族宗教、防震减灾、关心下一代等工作，支持发展妇女儿童、老龄、慈善和残疾人事业。

　　切实维护社会稳定。高度重视和正确处理好人民内部矛盾，制订发展措施、出台政策决策、确定发展项目都要注重从全局出发，做到公平公正，充分考虑人民群众的根本利益，努力从源头上预防和减少不稳定因素。坚决克服征地拆迁、土地流转、企业改制等工作中侵害群众利益的行为，切实维护好群众的合法权益。强化基层基础工作，引导广大群众理解和支持改革发展大局；进一步落实信访工作责任制，及时合理地解决好群众反映的问题。深化“平安常州”建设，加强社会治安综合治理，依法惩处各种违法犯罪活动。继续整顿和规范市场经济秩序，深入开展专项整治工作。高度重视安全生产管理和监督，防止重特大安全事故的发生。与此同时，认真办理人大代表建议和政协委员提案，充分发挥工会、共青团、妇联等人民团体的作用。

　　（八）继续为民办好实事，不断提高城乡居民生活质量

　　坚持把提高人民生活水平作为政府工作的出发点和落脚点，创新理念机制，集中财力物力，突出解决重点难点问题，使广大人民群众在改革发展中得到更多实惠。

　　今年，我们将着力为民办好10个方面的实事：（1）对部分困难群众提供救助。建立农村困难群众医疗救助制度，对城区的城市低保对象按人均300元标准筹措医疗救助资金；健全城乡低保标准年度增长机制，市区城市低保标准提高到每月260元；资助3000名贫困家庭学生完成学业。（2）建立以大病统筹为主的新型农村合作医疗保险制度，农民参保率达到80%以上。（3）全年新增城镇就业4万人，实现下岗失业人员再就业1.5万人，完成再就业培训3万人次。（4）改善居民居住条件。加快建设蓝天、丽华、陈渡桥、五星等经济适用房和定销商品房，完成120万平方米老小区的综合整治，解决150户“双特困”家庭的住房困难，改造拆迁危旧房1500户左右。（5）建设常州—金坛输水管道一期工程，使金坛市人民饮上长江水。（6）加快天然气利用步伐，全市13万煤气用户全部实施天然气置换。（7）增加城市绿化面积。新增城市公共绿地300公顷，建设东经120°公园、蔷薇湿地公园、青枫公园二期等一批绿化工程。（8）整治城市水环境。削减全市82家排污单位的水污染物排放总量，完成三井河等市河的综合整治。（9）实施农村客运班车通达工程，行政村客运班车通达率达到95%。（10）实施公交优先战略，新增公交车150辆、出租车200辆，新设一批公交线路和站点，与此同时，在城区部分区域和道路禁止摩托车通行。

　　三、切实加强政府自身建设

　　党的十六届四中全会对加强党的执政能力建设作出了全面部署，也对加强政府行政能力建设提出了新的要求。我们要不断创新发展理念、发展机制、发展能力和发展模式，逐步建立健全“领导形成核心、管理分清层次、决策按照程序、工作突出重点”的工作机制，全面提高领导经济社会发展的水平，努力建设法治政府、责任政府、廉洁政府和服务型政府。

　　增强审时度势的能力。正确分析和判断形势，是制定发展思路和对策的前提。深刻认识宏观经济发展趋势，准确把握宏观调控“有保有压”的精神实质，善于紧中见活、因势利导，大力推进高新技术产业、农业、服务业、城市基础设施和社会事业的发展；密切关注外部环境的新变化，善于趋利避害、正确应对，不断解决经济运行中出现的矛盾和困难；充分认识我市正处于工业转型期、城市化加速期和经济国际化提升期，善于扬长避短、乘势而上，认清自身经济社会发展中的闪光点，找准宏观调控与常州实际的结合点，占领加快发展、科学发展的制高点，实现我市经济社会发展的新跨越。

　　增强统筹发展的能力。统筹发展是科学发展观的根本要求。我们要始终坚持发展第一要务，不管遇到什么困难和挫折，都要紧紧围绕“两个率先”不动摇。始终坚持走科学发展之路，注重速度与结构、质量、效益的统一，推动城乡经济社会的良性互动，促进经济社会和人口、资源、环境协调发展，不断推进和谐社会建设。始终坚持走特色发展之路，充分发挥我市区位、交通、人文和自然资源等优势，把现有的、潜在的特色优势转化为竞争优势，不断增强我市的综合竞争力。始终坚持用创新的办法解决前进中的问题，妥善协调好各方面的利益，正确处理改革、发展与稳定的关系。同时，认真做好“十一五”规划的编制工作，科学描绘常州新一轮发展的蓝图，努力使“十一五”规划成为全面落实科学发展观、加快推进“两个率先”的战略规划。

　　增强科学决策的能力。大力推进决策的民主化、科学化，坚持重大事项的集体决策制、专家论证制。各级各部门要以对人民、对历史高度负责的态度，制定每一项政策、作出每一项决策、上每一个项目，都要切合实际，符合群众利益，对同群众利益密切相关的重大事项，实行公示、听证等制度，提高决策的透明度和公众的参与度。把握好决策的时机和策略，综合各方面的因素，做到敢于决策、善于决策、适时决策。建立决策责任追究制，对不按权限和程序决策造成重大损失的，要严肃追究责任。

　　增强真抓实干的能力。按照“做好每一件，干好每一天，抓好每一年”的要求，全体公务员特别是各级领导干部要敢于负责、勇挑重担，埋头苦干、少说多做。注重学习和研究经济规律、自然规律和社会发展规律，善于运用市场机制、市场主体整合配置各类资源，通过体制机制创新，努力做到少花钱甚至不花钱，也能办成事、多办事。提高依法行政的水平，严格按照法定权限和程序办事，不断规范和创新政府行政行为。提高实际操作能力，努力克服大而划之、似是而非的浮夸作风，对政府确定的每一项决策，对人民群众的每一项承诺，都要深入基层，了解实情，研究制定具体实施方案和政策措施，推动工作落实。进一步完善和加强政府目标管理，加大督查力度，按照“注重实绩，突出一线”的原则，建立科学有效的考核体系和奖惩办法，充分调动各方面的积极性。

　　增强拒腐防变的能力。坚持从严治政、廉洁从政，全面落实党风廉政建设责任制。健全以廉政准入制度为重点的制度防腐体系，努力从源头上预防和治理腐败。加大查处违法违纪案件力度，依法惩处腐败行为。大力弘扬艰苦奋斗、勤俭节约的优良传统，坚决遏制铺张浪费、公款吃喝、奢侈攀比之风。进一步完善政务公开制度，切实加大行政监察、审计监督的力度，加强社会监督。继续深入开展专项治理，纠正部门和行业的不正之风。加强廉洁自律教育，建设一支政治上靠得住、工作上有本事、作风上过得硬的公务员队伍。

　　各位代表，新的一年，我们深感肩负的责任重大而光荣，人民群众对政府的信任和支持，给了我们无穷的力量和必胜的信心。让我们在省委、省政府和市委的领导下，高举邓小平理论和“三个代表”重要思想伟大旗帜，紧紧依靠全市人民，上下一心，求真务实，开拓创新，为全面完成今年各项任务、夺取改革开放和现代化建设的新胜利而努力奋斗！
